# Anthropometric Estimations for Iranian General Population

**Published:** 2019-08

**Authors:** Mahnaz SAREMI, Mahnaz KAZEMHAGHIGHI

**Affiliations:** 1. Workplace Health Promotion Research Center, Shahid Beheshti University of Medical Sciences, Tehran, Iran; 2. Department of Ergonomics, School of Public Health and Safety, Shahid Beheshti University of Medical Sciences, Tehran, Iran

**Keywords:** Anthropometry, Estimation, Iran

## Abstract

**Background:**

An essential requirement exists for a single exhaustive source of anthropometric databank in Iran. Available information about Iranian bodily dimensions is not applicable to the general population due to the sample of people investigated. This study aimed to present the first Iranian anthropometric databank by estimation.

**Methods:**

After a systematic review, 24 relevant sources of information were found and included. No time limit was considered. The method of Rapid Anthropometrics Scaled for Height was used.

**Results:**

Overall, 36 bodily dimensions were estimated, for which the seven percentiles of 1^st^, 5^th^, 25^th^, 50^th^, 75^th^, 95^th,^ and 99^th^ were calculated, stratified by sex.

**Conclusion:**

The resulting tables can be claimed as the most representative anthropometric databank for Iranian general 20–64 yr population now. Data are suitable for practical purpose and are applicable in both occupational and community setting.

## Introduction

The main principle of ergonomics is to design the activity to match the characteristics of the user. On the other word, if an instrument, a workplace or a system is intended for human use, then its design should be based upon the characteristics of its human users. This principle, so-called “user-centered design”, could result in many enhancements in terms of functional efficiency, comfort, health, safety and quality of life (1). In contrast, the lack of incorporating anthropometric information in the design phase would result in an increase in the frequency of work-related injuries, as well as a decrease in human performance and well-being.

However, human beings are not all the same. Their anthropometric (e.g. body size, shape strength, and endurance), physiological, Biomechanical and psychological characteristics differ from one to another. In addition, factors such as age, sex, race, job, diet, physical exercise and so on influence human body dimensions (1, 2). These variabilities need to be taken into account by designers in order to provide adequate adjustability of workstations, tools, products and human-machine interfaces.

Because of the above mentioned human inter and intra-individual changes, the majority of developed and developing countries have produced their own anthropometric databank. Some examples include anthropometric data of Asian (3, 4), African (5, 6), European (7, 8) and American (9, 10) peoples. However, although publication of the first systematic anthropometric tables dated on 1950s, no anthropometric survey has yet been conducted on Iranian general population with regard to the occupational health application. Available data on this topic is mainly limited to Iranian industrial (11) and army (12) personnel which would not be presentative for the general population. Apart from its vital importance for designing various work stations and spaces, national anthropometric tables are required to fabricate any ease of use urban spaces such as public buildings, leisure facilities, general transportation services, and so on.

Since anthropometric surveys are often costly and time-consuming, ergonomists prefer to prepare anthropometric databases based on more simple methods such as estimation rather than measurement. One of the most widely employed methods of estimation is that proposed by Barkla (13) and Roebuck et al. (14). Entitled “Rapid Anthropometrics Scaled for Height” (RASH) by Pheasant, this method was validated and employed to estimate British anthropometric database (15, 16). The RASH method requires only data on the stature (i.e. mean and standard deviation) of an unknown population to which scaling factors derived from a known homogeneous population is applied. The technique was used in some similar researches afterward all over the world (17, 18).

An important need for appropriate anthropometric source on Iranian body characteristics exists among national ergonomists and designers. Recently, existed anthropometric data for Iranian population is not sufficiently comprehensive with respect to both sample size and representativeness (19). Therefore, the present study is the first attempt to provide a single comprehensive and representative source of anthropometric information on Iranian general population. Such data-bank is extensively applicable as a key element to provide ergonomic design requirements and to create products, hand tools, furniture, workstations, etc. as much fit as possible to the nation. In this regard, after an integrated review of related published literature, a dataset is assembled using the RASH method assumed to be sufficiently exhaustive and accurate for practical purpose; especially in terms of the creation of spaces in various public or industrial environments.

## Materials and Methods

The method of RASH was applied for estimating anthropometric database of Iranian general population. This method is based on the assumption that although people vary greatly in size, they are likely to be similar in proportions (2). It requires only know the mean (m) and standard deviation (s) of stature of an unknown population (i.e. target population). Scaling factors for intended bodily dimensions would be calculated from a known population (i.e. reference population) and then applied to the height data in the target one. Coefficient e_m_ was calculated using following formula (15):

$${\text{e}}_{\text{m}}=\frac{\bar{\text{x}}}{\bar{\text{h}}}$$

Where x̄ is the mean value of the intended bodily dimension and h̄ is the mean stature in the reference population.

Then, scaling ratio (E_m_) was obtained as arithmetic mean of e_m_ (15, 16):

$${\text{E}}_{\text{m}}=\frac{\sum \text{em}}{\text{n}}$$

The same method was used for estimating coefficients (e_s_) and scaling ratios (E_s_) related to the standard deviations. Calculations were made as a function of sex.

### The reference population

Conducting a systematic review using Google Scholar, Medline, SID, IranMedex, Magiran, MedLib and Civilica, an integrative collection of ever published anthropometric datasets of Iranian adults was developed. No time limit was considered. This collection was considered as the reference population. Studies were included as a function of their aim and methodology. Therefore, cases hypothesized any relationship between body variables (e.g. weight, BMI, wrist circumferences, etc.) and health problems (obesity, diseases, etc.) or conducted on children were excluded. Repeated datasets (i.e. same data published in more than one source) were also removed. As presented in Table 1, altogether 24 sources of information were found.

**Table 1:** The reference population

	***References***	***Sample size***	***Study population***
1	Shahnawaz and Davies (39)	400	Iranian steel workers
2	Mououdi (21)	179	University students
3	Abarghouie and HoseiniNasab (22)	330	Iranian adults
4	Motamedzade et al. (38)	303	Iranian office workers
5	Sadeghi and Habibi (23)	95	Bus drivers
6	Vafaee et al. (24)	115	University students
7	Mirmohammadi et al. (46)	911	University students
8	Mohammadi et al. (26)	70	Iranian women
9	Shokoohi H, Khoshroo (40)	853	Military personnel
10	Osquei-Zadeh et al. (25)	267	University students
11	Habibi et al. (47)	768	University students
12	Abedini et al. (27)	194	University students
13	Mououdi (28)	178	Iranian men
14	Mohammadi et al. (29)	140	Iranian women
15	Hemmatjoo et al. (30)	80	Military personnel
16	Falahati et al. (31)	70	University students
17	Ilbeigi et al. (32)	120	Iranian men
18	Davoudiantalab et al. (33)	400	Iranian male workers
19	Baharampour et al. (34)	194	University students
20	Pourtaghi et al. (12)	12635	Military personnel
21	Moshkdanian et al. (35)	300	Iranian adults
22	Eftekhar Vaghefi et al. (36)	1599	Medical personnel
23	Famil Alamdar and Famil Alamdar (37)	144	Iranian adults
24	Sadeghi et al. (11)	3720	Iranian workers

### The target population

For the target population, we have selected recent nationwide surveillance on noncommunicable disease risk factors, which in our knowledge is the best representative of the general population in terms of sample size, age, sex, socio-economic, and geographical distribution. Using a random multistage cluster sampling method, the study measured, among other variables, stature of 79,611 Iranian rural and urban citizens (50.1% men; 49.9% women) aged from 20 to 64 yr with standardized and calibrated instruments (20).

## Results

Scaling factors for 36 anthropometric estimations are presented in Table 2. Accordingly, stature has the highest ratios with eye and shoulders heights; and the smallest ratios with hand and foot breadths. Indeed, body dimensions of men and women are likely to follow a similar scaling profile (Table 2).

**Table 2:** Scaling ratios for mean (E_m_) and standard deviation (E_s_) of 36 bodily dimensions

	***Dimensions***	***Women***			***Men***		
		***E_m_***	***E_s_***	***n***	***E_m_***	***E_s_***	***n***
1	Stature	1.000	1.000	7	1.000	1.000	10
2	Eye height	0.928	1.224	7	0.936	1.021	9
3	Shoulder height	0.829	1.274	7	0.835	0.966	9
4	Elbow height	0.624	1.117	7	0.629	0.764	10
5	Hip height	0.540	1.165	4	0.533	0.855	5
6	Knuckle height	0.421	0.985	4	0.458	0.582	4
7	Fingertip height	0.391	0.746	5	0.400	0.585	6
8	Sitting height	0.531	0.779	7	0.581	0.685	11
9	Sitting eye height	0.467	0.897	6	0.521	0.667	10
10	Sitting elbow height	0.151	0.753	8	0.152	0.464	10
11	Thigh thickness	0.087	0.538	6	0.086	0.330	8
12	Sitting shoulder height	0.374	0.911	7	0.360	0.444	6
13	Buttock knee length	0.350	1.025	7	0.337	0.524	9
14	Buttock to popliteal length	0.280	0.924	8	0.271	0.499	11
15	Knee height	0.306	0.604	8	0.306	0.519	10
16	Popliteal height	0.251	0.608	10	0.246	0.451	11
17	Shoulder breadth (bi-deltoid)	0.251	0.495	10	0.258	0.452	12
18	Shoulder breadth (bi-acromial)	0.199	0.897	3	0.198	0.588	3
19	Hip breadth	0.224	0.562	9	0.207	0.449	10
20	Chest depth	0.150	0.462	8	0.131	0.508	9
21	Abdominal depth	0.157	0.917	6	0.134	0.527	8
22	Shoulder elbow length	0.208	0.681	8	0.212	0.443	8
23	Elbow fingertip length	0.262	0.466	4	0.257	0.590	4
24	Upper limb length	0.446	0.784	4	0.472	0.864	4
25	Shoulder grip length	0.373	0.816	5	0.368	0.855	4
26	Head length	0.113	0.638	4	0.110	0.460	5
27	Head breadth	0.087	0.540	4	0.087	0.253	5
28	Hand length	0.107	0.459	4	0.109	0.172	5
29	Hand breadth	0.044	0.245	5	0.048	0.118	6
30	Foot length	0.145	0.229	6	0.151	0.210	7
31	Food breadth	0.053	0.183	6	0.054	0.124	7
32	Vertical grip reach (standing)	1.198	2.525	4	1.215	1.171	5
33	Vertical grip reach (sitting)	0.718	0.787	3	0.809	0.783	4
34	Forward grip reach	0.420	1.052	5	0.440	1.020	4
35	Span	0.254	0.533	2	0.271	0.412	2
36	Elbow span	0.257	0.726	2	0.269	0.568	3

n=Number of available sources in each sex category;

Tables 3 and 4 show anthropometric estimates calculated based on these scaling factors for men and women, respectively. Iranian men’s average height is estimated to be 1697 mm versus 1564 mm for female. The tallest Iranian man is about 348 mm taller than the shortest one; while the tallest Iranian woman is about 317 mm taller than the shortest woman is.

**Table 3:** Anthropometric estimates for Iranian male adults (all dimensions in mm)

	***Dimensions***	***1th***	***5th***	***25th***	***50th***	***75th***	***95th***	***99th***	***SD***
1	Stature	1523	1574	1647	1697	1747	1820	1871	75
2	Eye height	1410	1462	1536	1588	1639	1713	1765	76
3	Shoulder height	1248	1297	1368	1416	1465	1535	1585	72
4	Elbow height	935	974	1030	1068	1106	1162	1201	57
5	Hip height	756	800	862	905	948	1010	1054	64
6	Knuckle height	676	706	748	777	807	849	879	44
7	Fingertip height	577	607	650	679	709	751	781	44
8	Sitting height	866	901	951	986	1020	1070	1105	51
9	Sitting eye height	768	802	851	884	918	966	1000	50
10	Sitting elbow height	177	201	235	258	281	315	339	35
11	Thigh thickness	89	106	130	147	163	187	204	25
12	Sitting shoulder height	533	556	588	611	633	665	688	33
13	Buttock knee length	481	508	546	572	598	637	663	39
14	Buttock to popliteal length	372	398	434	459	484	521	546	37
15	Knee height	429	455	493	519	545	583	610	39
16	Popliteal height	339	362	395	418	440	473	496	34
17	Shoulder breadth (bi-deltoid)	359	382	415	438	461	494	517	34
18	Shoulder breadth (bi-acromial)	234	264	307	337	366	409	439	44
19	Hip breadth	273	296	329	351	374	407	430	34
20	Chest depth	134	160	197	223	248	285	311	38
21	Abdominal depth	136	163	201	227	254	292	319	39
22	Shoulder elbow length	283	306	338	360	382	415	437	33
23	Elbow fingertip length	334	364	407	437	467	510	540	44
24	Upper limb length	650	694	757	801	844	907	951	65
25	Shoulder grip length	476	520	582	625	668	730	774	64
26	Head length	106	130	164	187	210	243	267	34
27	Head breadth	103	116	135	147	160	178	191	19
28	Hand length	155	163	176	185	193	206	214	13
29	Hand breadth	61	67	76	81	87	96	102	9
30	Foot length	220	231	246	257	267	283	293	16
31	Food breadth	71	77	86	92	98	107	114	9
32	Vertical grip reach (standing)	1859	1919	2004	2063	2122	2207	2267	88
33	Vertical grip reach (sitting)	1237	1277	1334	1373	1412	1469	1509	59
34	Forward grip reach	569	621	695	747	798	872	924	76
35	Span	388	410	440	460	481	511	532	31
36	Elbow span	358	387	428	457	485	526	555	43

**Table 4:** Anthropometric estimates for Iranian female adults (all dimensions in mm)

	***Dimensions***	***1th***	***5th***	***25th***	***50th***	***75th***	***95th***	***99th***	***SD***
1	Stature	1405	1452	1518	1564	1609	1676	1722	68
2	Eye height	1258	1315	1396	1452	1508	1589	1646	83
3	Shoulder height	1095	1154	1238	1296	1354	1439	1498	87
4	Elbow height	799	851	925	976	1027	1101	1153	76
5	Hip height	659	713	791	844	897	974	1028	79
6	Knuckle height	502	548	613	658	703	769	814	67
7	Fingertip height	494	529	578	612	646	696	730	51
8	Sitting height	706	742	794	830	865	917	953	53
9	Sitting eye height	589	630	690	731	772	831	873	61
10	Sitting elbow height	117	152	202	236	270	320	355	51
11	Thigh thickness	50	75	111	135	160	196	221	37
12	Sitting shoulder height	440	483	543	585	626	687	729	62
13	Buttock knee length	385	433	501	548	594	662	710	70
14	Buttock to popliteal length	292	335	396	438	480	542	585	63
15	Knee height	382	410	450	478	505	545	574	41
16	Popliteal height	297	325	365	393	421	461	489	41
17	Shoulder breadth (bi-deltoid)	314	337	370	393	415	448	471	34
18	Shoulder breadth (bi-acromial)	170	211	271	312	353	412	454	61
19	Hip breadth	261	287	324	350	375	413	439	38
20	Chest depth	162	183	214	235	256	287	308	31
21	Abdominal depth	100	142	203	245	287	347	390	62
22	Shoulder elbow length	218	250	295	326	357	402	434	46
23	Elbow fingertip length	335	357	388	409	430	461	483	32
24	Upper limb length	573	610	662	697	733	785	822	53
25	Shoulder grip length	453	491	545	583	620	674	712	56
26	Head length	76	105	148	177	206	248	278	43
27	Head breadth	51	76	112	136	161	197	222	37
28	Hand length	95	116	147	168	189	219	240	31
29	Hand breadth	30	41	58	69	80	96	108	17
30	Foot length	190	200	216	226	236	252	262	16
31	Food breadth	53	62	74	83	91	103	112	12
32	Vertical grip reach (standing)	1474	1591	1758	1874	1989	2156	2273	172
33	Vertical grip reach (sitting)	999	1035	1088	1124	1159	1212	1248	54
34	Forward grip reach	489	538	608	656	704	774	823	72
35	Span	312	337	372	397	421	456	481	36
36	Elbow span	287	321	369	402	435	484	517	49

## Discussion

The main purpose of the present study was to estimate as much as comprehensive and accurate anthropometric database for Iranian adults which could be applicable in industrial and nonindustrial design. Assembling the totality of relevant published tables by means of the simple, rapid and valid method of RASH, this study was able to present the first single source of anthropometric information for Iranian general population. It is therefore not illogical to claim that the present set of estimations is the most valid representation of the anthropometrics of the Iranian general 20–64 yr people achieved now. The pioneer in using this method was Pheasant, who developed an anthropometric source for British civilian adults based on a combination of the main previously published datasets (15).

For being representative, a sample should be an unbiased indication of the intended population. In the case of previously reported Iranian anthropometric datasets, one of the limitations face to the representativeness of data is that the sample size for about 80% of them is under 500 (21–39). Moreover, being conducted on the specified groups of industrial (11, 33, 39) or army (12, 30, 40) employees or in a specified location (32, 35), not across Iran, is the fact supporting the inaccuracy of using previous datasets for the general population. Anthropometric dimensions significantly differ between various occupational groups (41). Incorrect design of workplaces and products due to the lack of having access to an appropriate source of anthropometric databank could cause work-related physiological damages because of prolonged exposure to awkward postures. This could at least partly explain the high prevalence of musculoskeletal disorders in different Iranian industrial and nonindustrial sectors (42–45). Anthropometric dimensions should also be taken into consideration in the design of urban spaces such as public buildings, restaurants, hospitals and so on in order to provide an environment that supports the majority of residents especially with respect to some aspects such as clearance and reach.

Some key anthropometric dimensions are “knee height”, “sitting height” and “arms reach” (1).

A good anthropometric database should also be up-to-date. This feature is essential since human body characteristics vary over time and from generation to another. Our proposed set of estimations has the potential of being rapidly updated as soon as a more recent source of Iranian height would be available. Indeed, these data could be easily repeated for any sub-group of the general population.

Errors associated with using this technique are small and would be considered as negligible, even in comparison with common interpretation errors or those arising from the corrections for shoes and cloths (16). However, one could suggest that this method is much better applicable to body dimensions which best depend on the length of bones than circumferential dimensions. If relevant, this may be considered as a limitation of this study.

## Conclusion

Even though estimated data should be employed with prudence, but data prepared with this method is sufficiently reliable for many purposes (15). The application of the present anthropometric databank would be beneficial to better match the numerous manmade products and spaces with individual users. Therefore, a better match between national designs and Iranian users; as well as a more accurate evaluation of all products, machinery and spaces, either national or imported international ones, is expected. By means of integrating the presented tables into design phase, we hope national designers to provide greater safety, satisfaction and commonwealth for Iranian citizens.

## Ethical considerations

Ethical issues (Including plagiarism, informed consent, misconduct, data fabrication and/or falsification, double publication and/or submission, redundancy, etc.) have been completely observed by the authors.
